# Prevalence and Associated Factors of Poor Sleep Quality Among ‎adults in Qatar

**DOI:** 10.2147/NSS.S559592

**Published:** 2026-01-20

**Authors:** Mueen Al Ansi, Ayman Al-Dahshan, A A Alyafei, Mohamad Chehab, Sami Abdeen, Hanan Khudadad, Nada Adil, Mohamed Aabdien, Mohamed Iheb Bougmiza

**Affiliations:** 1 Preventive Medicine Division, Department of Medicine, Hamad Medical‎ Corporation, Doha, Qatar; 2 Preventive Health Department, Primary Health Care Corporation, Doha, Qatar; 3 Community Medicine Residency Program, Primary Health Care Corporation, Doha, Qatar; 4 Research Department, Primary Health Care Corporation, Doha, Qatar; 5 College of Medicine, Qatar University, Doha, Qatar

**Keywords:** sleep quality, Pittsburgh Sleep Quality Index, primary health care, stress

## Abstract

**Background:**

Poor sleep quality is increasingly recognized as a global public health concern linked to adverse physical, psychological, and occupational outcomes. However, data on sleep quality and its determinants remain scarce in Qatar.

**Objective:**

This study aimed to assess the prevalence of poor sleep quality and identify its associated factors among adults attending primary healthcare centers in Qatar.

**Methods:**

A cross-sectional study was conducted in six randomly selected primary health centers representing Qatar’s three health regions. Adults aged ≥18 years were recruited using multistage sampling. Data was collected through structured telephone interviews using validated tools, including the Pittsburgh Sleep Quality Index (PSQI), Perceived Stress Scale (PSS-4), GAD-2, PHQ-2, Sleep Hygiene Index, and IPAQ-short. Poor sleep quality was defined as PSQI ≥5. Multivariable logistic regression was performed to identify independent predictors.

**Results:**

Among 390 participants, 60.3% were classified as having poor sleep quality. Bivariate analysis showed significant associations between poor sleep and several factors, including female gender, Arab nationality, employment status, chronic disease, tobacco users, anxiety, depression, high perceived stress, and poor sleep hygiene. In the multivariable analysis, independent predictors of poor sleep were smokers (AOR=2.57, 95% CI: 1.43–4.64), higher perceived stress (AOR=1.1 per unit increase, 95% CI: 1.01–1.19), and higher Sleep Hygiene Index scores (AOR=1.06 per unit increase, 95% CI: 1.03–1.1).

**Conclusion:**

Poor sleep quality affects a substantial proportion of adults in Qatar and is significantly associated with modifiable lifestyle and psychological factors. Primary care-based interventions targeting stress management and sleep hygiene promotion are warranted.

## Introduction

Sleep is a vital physiological process essential for maintaining optimal physical health, emotional regulation, cognitive performance, and overall quality of life.^1^ Growing evidence indicates that insufficient or poor sleep quality is not only common but also a key contributor to a wide array of adverse health outcomes, including cardiovascular disease, type 2 diabetes, obesity, impaired immunity, depression, anxiety, and reduced productivity.^2^

In addition, poor sleep quality can lead to several health implications. It impairs cognitive and psychomotor functioning, such as thinking, mood, concentration, learning, memory, reaction time, and vigilance.^3^,^4^ As a result, sleep disturbances impact an individual’s wellbeing, safety, and productivity. It can also lead to injury or death from traffic and workplace accidents.^5^ Similarly, it is shared by scholars that inadequate sleep is associated with hypertension, diabetes, obesity, and cardiovascular disease.^6^,^7^

In recent decades, the prevalence of sleep disturbances has been rising worldwide, attributed to factors such as increased screen time, shift work, psychosocial stress, sedentary lifestyles, and unhealthy sleep environments.^8^ While sleep disorders are well-documented in many high-income countries, there remains a significant knowledge gap in the Middle East and North Africa region, ‎where cultural and social factors may play an important role in sleep health‎.^9^

In the region, ‎ a cross-sectional study among university students in Kuwait identified that three-quarters had ‎ poor sleep quality.^10^ Another study on the prevalence of poor sleep quality among diabetic ‎patients in Jizan, Saudi Arabia^11^ found that almost half are affected.^12^ In addition, a community-based ‎survey about diabetic Emiratis determined that nearly a third had poor sleep quality.^11^ A ‎study on the mobile usage and sleep quality among Saudi Arabian university students revealed ‎that more than a third suffered from poor sleep quality.^13^

The World Health Organization has increasingly emphasized the importance of sleep as a public health priority, particularly considering modern societal and occupational pressures that challenge healthy sleep patterns.^14^ Cultural, social, and environmental determinants may uniquely affect sleep quality in this region. For instance, several factors have been associated with poor sleep quality. These include sociodemographic characteristics such as employment status, income level, and marital status; the presence of comorbidities, particularly those related to mental health;^15^ behavioral factors such as physical inactivity, unhealthy dietary habits, and excessive use of mobile devices; as well as lifestyle practices like poor sleep hygiene and inadequate exposure to sunlight.^16^,^17^

Despite growing global recognition of the burden of poor sleep, no previous studies in Qatar have comprehensively examined the prevalence and correlates of poor sleep quality using validated tools. Moreover, the interaction between sleep quality and modifiable lifestyle factors remains underexplored in this population. To address this gap, the present study aimed to estimate the prevalence of poor sleep quality among adults attending primary healthcare centers in Qatar and to identify its associated sociodemographic, lifestyle, and psychological factors.

## Methods

### Study Design and Setting

An analytical cross-sectional study was conducted across six randomly selected primary health centers in Qatar, representing the three main health regions (Northern, Western, and Central). These centers, governed by the Primary Health Care Corporation (PHCC), serve a diverse population and provide subsidized or free healthcare services, making them representative of the broader adult population in Qatar.

### Study Population and Sampling

The study targeted adults (≥18 years) registered with PHCC who could communicate in either Arabic or English. Individuals who were unable to provide informed consent were excluded. Amultistage sampling technique was employed. First, two centers were randomly selected from each region. Then, within each selected center, participants were randomly chosen in proportion to the center’s population size. Eligible individuals were contacted by phone, informed about the study, and invited to participate.

### Sample Size

Assuming a 50% prevalence of poor sleep quality, a 5% margin of error, and a 95% confidence level, the estimated sample size was 384. A 50% prevalence was assumed because no prior national data on sleep quality in Qatar ‎were available. This provides the maximum sample size at a given confidence level‎.

### Data Collection Tool

Data were collected using a structured, interview-administered questionnaire that included:

- **Sociodemographic information.**
- **Sleep environment and habits.**
- **Psychological wellbeing:** assessed using the *Perceived Stress Scale-4* (PSS-4), *Generalized Anxiety Disorder-2* (GAD-2), and *Patient Health Questionnaire-2* (PHQ-2). Scores ≥ 3 on GAD-2 or PHQ-2 indicate possible anxiety or depression.
- **Sleep hygiene:** measured with the *Sleep Hygiene Index*, a 13-item scale (score range 13–65); higher scores denote poorer hygiene.
- **Physical activity:** evaluated with the *International Physical Activity Questionnaire-Short Form* (IPAQ-SF), classifying activity as low, moderate, or high.
- **Sleep quality:** assessed with the *Pittsburgh Sleep Quality Index* (PSQI), 19 items across seven domains; global scores > 5 indicate poor sleep quality.

All instruments have been validated previously, and internal consistency in this study was acceptable (Cronbach’s α: PSQI = 0.78, PSS-4 = 0.74, GAD-2 = 0.80, PHQ-2 = 0.82, SHI = 0.85).

Validated Arabic and English versions were used where available. A pilot test with 15 participants was conducted to assess clarity and estimate response time; necessary modifications were made accordingly.

### Data Collection Procedure

Trained data collectors conducted telephone interviews after obtaining verbal informed consent. Standardized procedures were followed to ensure consistency and confidentiality. All questionnaires were administered by phone due to ‎COVID-19 restrictions.

### Data Management and Analysis

Data were entered and cleaned by the principal investigator using SPSS version 28. Descriptive statistics were used to summarize variables. The chi-square test and independent *t*-test were used to examine associations between categorical and continuous variables, respectively. Binary logistic regression was performed to identify independent predictors of poor sleep quality (defined as PSQI ≥5). Missing or incorrectly collected data were excluded from the analysis, and participant recruitment continued until the required sample size was achieved. A p-value ≤0.05 was considered statistically significant.

### Ethical Considerations

Ethical approval for this study was obtained from the Research Department of Primary Healthcare Corporation (PHCC) (reference number PHCC/DCR/2020/04/027). This study was conducted in accordance with the ethical standards laid down in the Declaration of Helsinki.

As the study was conducted entirely through telephone interviews during the late phase of the COVID-19 pandemic, written informed consent was not feasible. Instead, verbal informed consent was obtained from all participants, a procedure that was reviewed and approved by the PHCC Research Department.

Each instance of verbal consent was documented by the data collectors in a standardized consent log, which included the participant’s study identification code, the date and time of consent, and the initials of the data collector who obtained it. Data confidentiality was ensured throughout the study, and referrals were recommended for participants who exhibited signs of poor mental health or sleep disorders.

## Results

Table 1 presents the sociodemographic characteristics of the 390 adult participants. The majority were aged 30–39 years (41.8%), female (53.3%), and had attained a college-level education or higher (76.9%). Most participants were married (74.4%), of Arab nationality (65.1%), and employed (75.6%). Egyptians constituted the largest national subgroup (22.3%).

**Table 1:** Sociodemographic Characteristics of Study Participants (N=390)

Characteristic		Frequency (%)
**Age** (years)	Less than 30	88 (22.6)
	30-39	163 (41.8)
	40-54	104 (26.7)
	55 or older	35 (9)
	Mean±SD	37.28±10.22
**Gender**	Male	182 (46.7)
	Female	208 (53.3)
**Educational level**	Preparatory school or lower	21 (5.4)
	Secondary school	69 (17.7)
	College or higher	300 (76.9)
**Marital status**	Not married	100 (25.6)
	Married	290 (74.4)
**Nationality**	Egypt	87 (22.3)
	Sudan	50 (12.8)
	Jordan	55 (14.1)
	India	33 (8.5)
	Philippines	37 (9.5)
	Others	128 (32.8)
	Arab	254 (65.1)
	Non-Arab	136 (34.9)
**Employment status**	Unemployed	95 (24.4)
	Employed	295 (75.6)

**Notes**: Arab nationality includes participants originating from Middle Eastern and North African ‎countries (eg, Egypt, Sudan, Jordan, Lebanon, Syria, etc.).

**Abbreviation**: SD, standard deviation.

Table 2 summarizes lifestyle and health-related characteristics. A significant proportion of participants were overweight or obese (71.6%), with 33.8% classified as obese. Around 28.1% were engaged in shift work, and 28.2% reported having chronic diseases. About one-fifth of the sample (19.8%) were current smokers. Most participants did not meet recommendations for fruit/vegetable intake (72.6%) or physical activity (71.5%). Psychological distress was notable, with 41.3% screening positive for anxiety and 23.1% for depression. The mean perceived stress score was 5.9±3.0, and the mean Sleep Hygiene Index score was 21.5±13.6. Overall, 60.3% of participants reported poor sleep quality.

**Table 2:** Lifestyle Medicine-Related Characteristics of Study Participants (N=390)

Characteristic		Frequency (%)
**Obesity categories**	Normal	111 (28.5)
	Overweight	147 (37.7)
	Obesity class 1	86 (22.1)
	Obesity class 2	35 (9)
	Obesity class 3	11 (2.8)
**Obesity**	Non-obese	258 (66.2)
	Obese	132 (33.8)
**Shift works**	No	207 (71.9)
	Yes	81 (28.1)
**History of chronic disease**(**s**)	No	280 (71.8)
	Yes	110 (28.2)
**Current smoking**	No	312 (80.2)
	Yes	77 (19.8)
**Fruit and vegetables intake**	Not meeting the recommendation	283 (72.6)
	Meeting the recommendations	107 (27.4)
**Physical activity**	Not meeting the recommendation	279 (71.5)
	Meeting the recommendations	111 (28.5)
**Anxiety (GAD2)**	No Anxiety	229 (58.7)
	Anxiety	161 (41.3)
**Depression (PHQ2)**	No Depression	300 (76.9)
	Depression	90 (23.1)
**Perceived stress scale (**mean±SD)		5.9±3.0
**Sleep Hygiene index (**mean±SD)		21.5±13.6
**Sleep quality**	Good sleep	155 (39.7)
	Poor sleep	235 (60.3)

**Abbreviations**: SD, standard deviation; GAD-2, Generalized Anxiety Disorder Questionnaire −2; PHQ-2, Patient Health Questionnaire-2.

Table 3 explores the bivariate associations between participant characteristics and sleep quality. Poor sleep was significantly more prevalent among females (66.8%), Arabs (64.6%), unemployed individuals (72.6%), those with chronic diseases (70%), smokers (45.5%), and those reporting anxiety (68.3%), depression (74.4%), or high perceived stress (67.9%) (p<0.001). In addition, poor sleepers had significantly higher perceived stress and Sleep Hygiene Index scores compared to good sleepers (p<0.001).

**Table 3:** Relationship Between Participants’ Characteristics and Sleep Quality (N=390)

Characteristics		Sleep Quality (PSQI)		P-value
		Good Sleep n (%)	Poor Sleep n (%)	
**Age categories**	Less than 30	38 (43.2)	50 (56.8)	0.596
	30-39	68 (41.7)	95 (58.3)	
	40-54	37 (35.6)	67 (64.4)	
	55 or older	12 (34.3)	23 (65.7)	
**Gender**	Male	86 (47.3)	96 (52.7)	0.005
	Female	69 (33.2)	139 (66.8)	
**Educational level**	Primary school	7 (33.3)	14 (66.7)	0.813
	Secondary school	27 (39.1)	42 (60.9)	
	College or higher	121 (40.3)	179 (59.7)	
**Marital status**	Not married	43 (43)	57 (57)	0.440
	Married	112 (38.6)	178 (61.4)	
**Nationality**	Egypt	21 (24.1)	66 (75.9)	0.003
	Sudan	15 (30)	35 (70)	
	Jordan	26 (47.3)	29 (52.7)	
	India	18 (54.5)	15 (45.5)	
	Philippines	15 (40.5)	22 (59.5)	
	Others	60 (46.9)	68 (53.1)	
	*Arab*	90 (35.4)	164 (64.6)	
	*Non-Arab*	65 (47.8)	71 (52.2)	
**Employment status**	Unemployed	26 (27.4)	69 (72.6)	0.005
	Employed	129 (43.7)	166 (56.3)	
**BMI** mean±SD		27.61±5.03	28.57±5.69	0.086
**Obesity categories**	Normal	50 (45)	61 (55)	0.414
	Overweight	61 (41.5)	86 (58.5)	
	Obesity class 1	29 (33.7)	57 (66.3)	
	Obesity class 2	12 (34.3)	23 (65.7)	
	Obesity class 3	3 (27.3)	8 (72.7)	
**Obesity**	Non obese	111 (43)	147 (57)	0.064
	Obese	44 (33.3)	88 (66.7)	
**Current smoking**	No	199 (63.8)	113 (36.2)	0.003
	Yes	35 (45.5)	42 (54.5)	
**Shift works**	No	‎86 (41.5) ‎	‎121 (58.4)‎	‎0.163‎
	Yes	‎41 (50.6)‎	‎40 (49.4)‎	
**History of chronic disease**	No	122 (43.6)	158 (56.4)	0.014
	Yes	33 (30)	77 (70)	
**Fruit and vegetables intake**	Not meeting the recommendation	115 (40.6)	168 (59.4)	0.558
	Meeting the recommendations	40 (37.4)	67 (62.6)	
**Physical activity**	Not meeting the recommendation	104 (37.3)	175 (62.7)	0.114
	Meeting the recommendations	51 (45.9)	60 (54.1)	
**Anxiety (GAD2)**	No Anxiety	104 (45.4)	125 (54.6)	0.006
	Anxiety	51 (31.7)	110 (68.3)	
**Depression (PHQ2)**	No Depression	132 (44)	168 (56)	0.002
	Depression	23 (25.6)	67 (74.4)	
**Stress (PSS-4)**	No stress	87 (48.9)	91 (51.1)	0.001
	Stress	68 (32.1)	144 (67.9)	
**Perceived stress scale (**mean±SD)		5.12±2.83	6.35±3.03	<0.001
**Sleep Hygiene index (**mean±SD)		14.95±7.05	17.81±7.61	<0.001

**Notes**: p < 0.05 considered statistically significant.

**Abbreviations**: BMI, body mass index; SD, standard deviation; PSQI, Pittsburgh Sleep Quality Index; PHQ-2, Patient Health Questionnaire-2; GAD-2, Generalized Anxiety Disorder Questionnaire-2; PSS-4, Perceived Stress Scale Questionnaire −4.

Table 4 displays the results of multivariable logistic regression identifying predictors of poor sleep quality. Smoking (AOR=2.57, 95% CI: 1.43–4.64, p=0.002), higher perceived stress scores (AOR=1.1 per unit increase, 95% CI: 1.01–1.19, p=0.025), and higher Sleep Hygiene Index scores (AOR=1.06 per unit increase, 95% CI: 1.03–1.1, p=0.001) were independently associated with increased odds of reporting poor sleep quality.

**Table 4:** Predictors of Poor Sleep Among the Participants Using Multivariable Logistic Regression

Characteristic		AOR (95% CI)	P-value
**Age categories**	Less than 30	0.69 (0.26–1.81)	0.453
	30-39	0.92 (0.37–2.28)	0.854
	40-54	1.32 (0.53–3.26)	0.548
	55 or older	[Reference]	
**Gender**	Male	0.77 (0.47–1.27)	0.306
	Female	[Reference]	
**Nationality**	Arab	1.43 (0.88–2.33)	0.149
	Non-Arab	[Reference]	
**Employment status**	Unemployed	1.44 (0.78–2.66)	0.245
	Employed	[Reference]	
**Obesity**	Non obese	0.7 (0.43–1.14)	0.149
	Obese	[Reference]	
**History of chronic disease/s**	No	0.6 (0.34–1.05)	0.074
	Yes	[Reference]	
**Current smoking**	No	[Reference]	0.002
	Yes	2.57 (1.43–4.64)	
**Physical activity**	Not meeting the recommendation	1.43 (0.86–2.37)	0.172
	Meeting the recommendations	[Reference]	
**Anxiety (GAD2)**	No Anxiety	0.77 (0.48–1.26)	0.301
	Anxiety	[Reference]	
**Depression (PHQ2)**	No Depression	0.6 (0.33–1.1)	0.101
	Depression	[Reference]	
**Perceived stress scale**		1.1 (1.01–1.19)	0.025
**Sleep Hygiene index**		1.06 (1.03–1.1)	0.001

**Notes**: p < 0.05 considered statistically significant; [Reference] = baseline category in the regression model.

**Abbreviations**: AOR, adjusted odds ratio; CI, confidence interval; PSQI, Pittsburgh Sleep Quality Index; GAD2, Generalized Anxiety Disorder Questionnaire-2; PHQ2‎, Patient Health Questionnaire-2.

## Discussion

This study provides a comprehensive evaluation of sleep quality among adults attending primary healthcare centers in Qatar, utilizing validated instruments including the Pittsburgh Sleep Quality Index (PSQI), Sleep Hygiene Index, and standardized mental health screening tools. Our findings reveal a high prevalence of poor sleep quality, affecting approximately 60.3% of participants, and identify several key factors independently associated with this issue: current smoking, higher perceived stress, and poorer sleep hygiene practices.

Our finding of poor sleep quality is consistent with a recent meta-analysis in Southeast Asia, which showed a pooled prevalence of poor sleep quality of 64% (95% CI: 53–75%).^18^ Also, the observed prevalence of poor sleep quality aligns with findings from other countries in the Middle East and North Africa (MENA) region. For instance, studies conducted among visitors of Primary Healthcare Centers in Saudi Arabia and Kuwait reported poor sleep quality rates of around 72% and 60%, respectively.^19^,^20^

Similarly, ‎ a national study among Jordanian university students found that two-thirds described their ‎sleep as poor-quality ‎,^21^ further underscoring the regional importance of this public health issue. Compared to these studies, our population-based estimate among adults in Qatar confirms that sleep health is a significant yet underrecognized concern within the general population.

Consistent with previous literature, poor sleep quality in our study was more prevalent among females and unemployed individuals.^22^,^23^ These patterns may reflect the influence of gender-related psychosocial stressors and the association between unemployment and financial or psychological distress, which are known contributors to sleep disturbances.^24^

Furthermore, we identified significant bivariate associations with several modifiable lifestyle and mental health factors, including smoking, chronic disease history, anxiety, depression, and high perceived stress. Importantly, the multivariable logistic regression analysis highlighted three independent predictors of poor sleep quality. Current smokers were over twice as likely to report poor sleep compared to non-smokers (AOR=2.57). This finding aligns with existing evidence suggesting that nicotine use interferes with circadian rhythm and sleep architecture, reducing total sleep time and increasing arousals.^25^,^26^ It is also plausible that individuals experiencing poor sleep may use nicotine as a coping mechanism, pointing to a potentially bidirectional relationship that warrants further exploration.^27^

Higher perceived stress emerged as another strong predictor of poor sleep. This finding is consistent with other studies conducted in other countries.^28^,^29^ Psychological stress activates the hypothalamic-pituitary-adrenal (HPA) axis and increases cortisol levels, which can disrupt sleep initiation and maintenance.^30^ This relationship emphasizes the importance of stress management and mental health interventions as part of sleep health promotion efforts,^31^ particularly in high-demand occupational or social environments such as those found in Qatar.

Additionally, poor sleep hygiene practices were significantly associated with increased odds of poor sleep quality. Behaviors such as irregular sleep schedules, evening screen use, caffeine consumption before bedtime, and inappropriate sleep environments are well-established disruptors of healthy sleep.^32^,^33^

Our findings suggest that promoting good sleep hygiene habits could be an effective and low-cost intervention to improve sleep outcomes in this population. While several other factors showed significant associations with sleep quality in bivariate analysis—such as obesity, chronic disease, and mental health symptoms—these did not retain statistical significance in the multivariate model. This may reflect confounding effects or the interrelatedness of lifestyle and psychological factors. For example, stress and poor sleep hygiene may mediate the impact of chronic illness or depression on sleep outcomes.^34^ The absence of a significant association between chronic disease and sleep quality may also be explained by the relatively young age of our sample (mostly 30–39 years), who are less likely to have advanced chronic conditions that affect sleep. These interactions highlight the need for holistic and integrated approaches when addressing sleep health.

Data collection occurred during the late phase of the COVID-19 pandemic. This period may have temporarily influenced stress levels, routines, and sleep behaviors, leading to higher reported prevalence of poor sleep. While this context limits generalizability to post-pandemic conditions, it provides important insight into sleep health during a period of social and behavioral disruption.

The results of this study have important public health and clinical implications. Given the high burden of poor sleep and its associations with modifiable behaviors and stress, primary care settings offer a valuable opportunity for early identification and intervention.^35^,^36^

### Strength and Limitations

This study has several strengths, including its use of validated multidimensional ‎instruments, a ‎representative sample from diverse health centers across Qatar, and a ‎comprehensive ‎assessment of behavioral and psychological correlates of sleep quality. ‎However, several ‎limitations should be acknowledged. The cross-sectional design ‎precludes causal inference. ‎Additionally, reliance on self-reported data may introduce ‎recall and social desirability biases. Furthermore, the study was conducted during the late phase of the COVID-19 pandemic, when stress and behavioral patterns were atypical; therefore, prevalence estimates may not reflect post-pandemic sleep trends. Finally, potential selection bias due to telephone-based recruitment—may exclude night-shift workers or those with severe sleep disturbance.

## Conclusions

This study reveals a high prevalence of poor sleep quality among adults attending primary healthcare centers in Qatar, with over 60% of participants affected. Key independent predictors included current smoking, elevated perceived stress, and poor sleep hygiene practices. These findings highlight the need for integrated, preventive approaches within primary care. In light of data collection during the COVID-19 period, the results should be interpreted with caution regarding temporal generalizability. Targeted interventions focusing on stress reduction and promotion of healthy sleep habits are essential to improve sleep quality and overall well-being in the adult population. Further research should include qualitative studies to understand cultural influences on sleep, and intervention trials to assess the impact of sleep hygiene and stress reduction programs in primary care.

## Data Availability

The datasets generated and/or analyzed during the current study are available from the corresponding author, Dr. Mueen Al Ansi (email: malansi@hamad.qa) upon reasonable request.

## References

[1] Ramar K, Malhotra RK, Carden KA, et al. Sleep is essential to health: an American academy of sleep medicine position statement. *J Clin Sleep Med*.;17(10):2115–11. doi:10.5664/jcsm.9476. PMC8494094 34170250

[2] Sleep Foundation. Effects of sleep deprivation [Internet]. Sleep Foundation. 2023. Available from: https://www.sleepfoundation.org/sleep-deprivation/effects-of-sleep-deprivation. Accessed January 09, 2026.

[3] Shah A, Ayas N, Tan WC, et al. Sleep quality and nocturnal symptoms in a community-based COPD cohort. *COPD*. 2020;17(1):40–48. doi:10.1080/15412555.2019.1695247 31920133 PMC7449828

[4] Zeng LN, Yang Y, Wang C, et al. Prevalence of poor sleep quality in nursing staff: a meta-analysis of observational studies. *Behav Sleep Med*. 2020;18(6):746–759. 31672062 10.1080/15402002.2019.1677233

[5] Lallukka T, Kronholm E. The contribution of sleep quality and quantity to public health and work ability. *Eur J Public Health*. 2016;26(4):532. doi:10.1093/eurpub/ckw049 27126826

[6] Matsuda R, Kohno T, Kohsaka S, et al. The prevalence of poor sleep quality and its association with depression and anxiety scores in patients admitted for cardiovascular disease: a cross-sectional designed study. *Int J Cardiol*. 2017;228:977–982. doi:10.1016/j.ijcard.2016.11.091 27915216

[7] Rodriguez-Gonzalez-Moro MT, Rodriguez-Gonzalez-Moro JM, Rivera-Caravaca JM, Vera-Catalan T, Simonelli-Munoz AJ, Gallego-Gomez JI. Work shift and circadian rhythm as risk factors for poor sleep quality in public workers from Murcia (Spain). *Int J Environ Res Public Health*. 2020;17(16):5881. doi:10.3390/ijerph17165881 32823687 PMC7459974

[8] Du M, Liu M, Wang Y, Qin C, Liu J. Global burden of sleep disturbances among older adults and the disparities by geographical regions and pandemic periods. *SSM*. 2023;25:101588. doi:10.1016/j.ssmph.2023.101588 PMC10788304 38225953

[9] Chaabane S, Chaabna K, Khawaja S, Aboughanem J, Mamtani R, Cheema S. Epidemiology of sleep disturbances among medical students in the Middle East and North Africa: a systematic review and meta-analysis. *J Global Health*. 2025;15. 10.7189/jogh.15.04099 PMC12023807 40277296

[10] Al-Kandari S, Alsalem A, Al-Mutairi S, Al-Lumai D, Dawoud A, Moussa M. Association between sleep hygiene awareness and practice with sleep quality among Kuwait University students. *Sleep Health*. 2017;3(5):342–347. doi:10.1016/j.sleh.2017.06.004 28923190

[11] Bani-Issa W, Al-Shujairi AM, Patrick L. Association between quality of sleep and health-related quality of life in persons with diabetes mellitus type 2. *J Clin Nurs*. 2018;27(7–8):1653–1661. doi:10.1111/jocn.14221 29266588

[12] Darraj A, Mahfouz MS, Alsabaani A, Sani M, Alameer A. Assessment of sleep quality and its predictors among patients with diabetes in Jazan, Saudi Arabia. *Diabetes Metab Syndr Obes*. 2018;11:. doi:10.2147/DMSO.S178674 PMC6163002 30288072

[13] Rafique N, Al-Asoom LI, Alsunni AA, Saudagar FN, Almulhim L, Alkaltham G. Effects of mobile use on subjective sleep quality. *Nat Sci Sleep*. 2020;12:357–364. 32607035 10.2147/NSS.S253375 PMC7320888

[14] Stranges S, Tigbe W, Gómez-Olivé FX, Thorogood M, Kandala NB. Sleep Problems: an emerging global epidemic? Findings from the INDEPTH WHO-SAGE study among more than 40,000 older adults from 8 countries across Africa and Asia. *SLEEP*. 2012;35(8):1173–1181. doi:10.5665/sleep.2012 22851813 PMC3397790

[15] Papadopoulos D, Sosso FAE. Socioeconomic status and sleep health: a narrative synthesis of 3 decades of empirical research. *J Clin Sleep Med*. 2022;19(3):605–620. doi:10.5664/jcsm.10336 PMC9978435 36239056

[16] Gupta CC, Duncan MJ, Ferguson SA, et al. Exploring the prioritisation of sleep, diet, and physical activity as pillars of health: correlates and associations with health behaviours in Australian adults. *J Activity Sedentary and Sleep Behav*. 2023;2(1). doi:10.1186/s44167-023-00035-3 PMC11960265 40217386

[17] Manzar MD, BaHammam AS, Hameed UA, et al. Dimensionality of the pittsburgh sleep quality index: a systematic review. *Health Qual Life Outcomes*. 2018;16(1):89. doi:10.1186/s12955-018-0915-x 29743066 PMC5944037

[18] Satriono FD, How SL, Islam TS, Wishwadewa WN, Habil MH. Prevalence of poor sleep quality based on Pittsburgh Sleep Quality Index (PSQI) among medical students in Southeast Asia: a systematic review and meta-analysis. *J Sleep Disord Manag*. 2024;9:046. doi:10.23937/2572-4053.1510046

[19] Albinsaleh AA, Al Wael WM, Nouri MM, Alfayez AM, Alnasser MH, Alramadan MJ. Prevalence and factors associated with poor sleep quality among visitors of primary healthcare centers in Al-Ahsa, Kingdom of Saudi Arabia: an analytical cross-sectional study. *cureus*.;15(7):e42653. PMID: 37644931; PMCID: PMC10461694. doi:10.7759/cureus.42653 PMC10461694 37644931

[20] Al-Rashed F, Sindhu S, Al Madhoun A, et al. Short sleep duration and its association with obesity and other metabolic risk factors in Kuwaiti Urban adults. *Nat Sci Sleep*. 2021;13:1225–1241. 34335063 10.2147/NSS.S311415 PMC8318215

[21] Albqoor MA, Shaheen AM. Sleep quality, sleep latency, and sleep duration: a national comparative study of university students in Jordan. *Sleep Breath*. 2021;25(2):1147–1154. doi:10.1007/s11325-020-02188-w 33034880

[22] Alosta MR, Oweidat I, Alsadi M, Alsaraireh MM, Oleimat B, Othman EH. Predictors and disturbances of sleep quality between men and women: results from a cross-sectional study in Jordan. *BMC Psychiatry*. 2024;24(1):200.PMID: 38475779; PMCID: PMC10936022. doi:10.1186/s12888-024-05662-x 38475779 PMC10936022

[23] Blanchflower Y, G D, Bryson A. Unemployment and sleep: evidence from the United States and Europe. *Econ Hum Biol*. 2021;43:101042. 34271429 10.1016/j.ehb.2021.101042

[24] Visvalingam N, Sathish T, Soljak M, et al. Prevalence of and factors associated with poor sleep quality and short sleep in a working population in Singapore. *Sleep Health*. 2020;6(3):277–287. doi:10.1016/j.sleh.2019.10.008 31836498

[25] Hwang JH, Park SW. The relationship between poor sleep quality measured by Pittsburgh Sleep Quality Index and cigarette smoking according to sex and age. *Epidemiol Health*. 2022;44:e2022022. doi:10.4178/epih.e2022022 35167741 PMC9117098

[26] Lee SY, Ju YJ, Lee JE, et al. Factors associated with poor sleep quality in the Korean general population: providing information from the Korean version of the Pittsburgh Sleep Quality Index. *J Affect Disord*. 2020;271:49–58. doi:10.1016/j.jad.2020.03.069 32312697

[27] Singh N, Wanjari A, Sinha AH. Effects of nicotine on the central nervous system and sleep quality in relation to other stimulants: a narrative review. *Cureus*. 2023. doi:10.7759/cureus.49162 PMC10733894 38130519

[28] Thomée S, Annika H, Mats H. Mobile phone use and stress, sleep disturbances, and symptoms of depression among young adults - a prospective cohort study. *BMC Public Health*. 2011;11:1–11. doi:10.1186/1471-2458-11-66 21281471 PMC3042390

[29] Nor Asma MUSA FMMa LPW. Prevalence and factors associated with poor sleep quality among secondary school teachers in a developing country. *Industrial Health*. 2018;208(56):407–30. 10.2486/indhealth.2018-0052 PMC6172183 29848899

[30] Herman JP, McKlveen JM, Ghosal S, et al. Regulation of the hypothalamic‐pituitary‐adrenocortical stress response. *Compr. Physiol*. 2016;6(2):603–621. 27065163 10.1002/cphy.c150015 PMC4867107

[31] Albakri U, Drotos E, Meertens R. Sleep health promotion interventions and their effectiveness: an umbrella review. *Int J Environ Res Public Health*. 2021;18(11):5533.PMID: 34064108; PMCID: PMC8196727. doi:10.3390/ijerph18115533 34064108 PMC8196727

[32] Segon T, Melkam M, Tinsae T, et al. Poor sleep hygiene practice and associated factors among adults with epilepsy attending follow up care at Mettu Karl comprehensive specialized hospitals in Illu Ababora Zone and general hospital in Buno Bedele zone, Southwest Ethiopia’. *Sleep Sci Pract*. 2024;8(1). doi:10.1186/s41606-024-00110-x

[33] Gokcen N, Komac A, Kuru FT, et al. Inadequate sleep hygiene as a key factor in poor sleep quality in systemic sclerosis: an observational, cross-sectional study. *Rheumatol Int*. 2025;45(2). doi:10.1007/s00296-025-05794-7. PMC11785615 39888416

[34] De Menezes-Júnior LAA, De Moura SS, Machado-Coelho GLL, Meireles AL. How anxiety and depression mediate the link between sleep quality and health perception during crisis periods. *Sci Rep*. 2025;15(1). doi:10.1038/s41598-025-98004-0 PMC12000303 40234587

[35] Awadalla NJ, Mahfouz AA, Shehata SF, et al. Sleep hygiene, sleep-related problems, and their relations with quality of life in a primary-care population in southwest Saudi Arabia. *J Family Med Primary Care*. 2020;9(6):3124–30. 32984184 10.4103/jfmpc.jfmpc_525_20 PMC7491796

[36] Chaput JP, Shiau J. Routinely assessing patients’ sleep health is time well spent. *Prev Med Rep*. 2019;14:100851. doi:10.1016/j.pmedr.2019.100851 30956942 PMC6434165

